# Past Life and Future Effects—How Heterologous Infections Alter Immunity to Influenza Viruses

**DOI:** 10.3389/fimmu.2018.01071

**Published:** 2018-05-22

**Authors:** Aisha Souquette, Paul G. Thomas

**Affiliations:** Department of Immunology, St. Jude Children’s Research Hospital, Memphis, TN, United States

**Keywords:** CD8+ T cells, influenza, heterologous, bystander, attrition, memory, cross-reactivity, chronic co-infection

## Abstract

Influenza virus frequently mutates due to its error-prone polymerase. This feature contributes to influenza virus’s ability to evade pre-existing immunity, leading to annual epidemics and periodic pandemics. T cell memory plays a key protective role in the face of an antigenically distinct influenza virus strain because T cell targets are often derived from conserved internal proteins, whereas humoral immunity targets are often sites of increased mutation rates that are tolerated by the virus. Most studies of influenza T cell memory are conducted in naive, specific pathogen free mice and do not account for repetitive influenza infection throughout a lifetime, sequential acute heterologous infections between influenza infections, or heterologous chronic co-infections. By contrast to these mouse models, humans often experience numerous influenza infections, encounter heterologous acute infections between influenza infections, and are infected with at least one chronic virus. In this review, we discuss recent advances in understanding the effects of heterologous infections on the establishment and maintenance of CD8+ T cell immunological memory. Understanding the various factors that affect immune memory can provide insights into the development of more effective vaccines and increase reproducibility of translational studies between animal models and clinical results.

## Introduction

### Influenza Virus

In the United States, seasonal epidemics caused by influenza virus lead to 3.1 million hospitalized days, 31.4 million outpatient visits, and direct medical costs of $10.4 billion, on average (1). While vaccination against influenza virus has decreased morbidity and mortality, influenza virus is particularly efficient at evading the immune system, and more research is needed to improve vaccine efficacy. A key aspect of influenza virus biology, which confers higher pathogenicity and contributes to immune evasion, is its high rate of mutation due to the error-prone activity of its RNA polymerase, which lacks proofreading function. Accrual of point mutations over time, known as antigenic drift, can lead to antigenically distinct proteins that cannot be recognized by established protective immunity. Evasion of immune memory can also occur when more than one parental virus strain infects the same host and reassortment of various genome segments leads to viral progeny of a new subtype, a process known as antigenic shift. Indeed, the 2009 H1N1 pandemic was the result of reassortment between an Eurasian swine H1N1 and a triple reassortant swine H1N2, which contained gene segments from an avian virus, North American classical swine H1N1, and human seasonal H3N2 (2).

Influenza virus is a member of the Orthomyxovirus family. There are three classes: A, B, and C; which vary in their host, pathogenicity, and structure. The genome consists of 7–8 segments of negative-sense single stranded RNA, encapsulated in nucleoprotein (NP). At the end of each segment is a heterotrimer of three polymerase proteins: polymerase basic protein 1, polymerase basic protein 2, and polymerase acidic protein (PA). The genome is enclosed in a capsid, which is encapsulated in a host derived lipid bilayer envelope. Imbedded into the lipid envelope is the matrix 2 protein, and two spike proteins that are important for binding, fusion/entry, and egress from target cells—hemagglutinin (HA) and neuraminidase (NA). Influenza viruses are often subdivided and referred to by their HA and NA subtypes. Of the 18 HA and 11 NA subtypes currently known, only H1N1, H2N2, and H3N2 have caused a human pandemic. Currently circulating human influenza viruses include: influenza A virus H1N1, influenza A virus H3N2, and influenza B virus.

Control of influenza virus is ultimately achieved by the virus-specific adaptive immune response. CD4+ T cells aid in the activation of both B and CD8+ T cells, important for production of antibodies and clearance of virus infected cells, respectively. Antibodies produced by B cells serve non-neutralizing and neutralizing functions. HA and NA are major targets of neutralizing antibodies; however, these proteins are often sites of mutations, which may lead to antigenic drift over time. Conversely, CD8 T cells, also known as cytotoxic T lymphocytes (CTLs), target conserved regions of internal proteins, which are less prone to mutation due to fitness cost and/or potential for loss of function. By contrast to innate immunity, adaptive immunity is pathogen-specific and results in immunological memory. In cases of antigenically distinct subtypes, which are common for influenza virus, and often lead to pandemics, targeting of conserved internal proteins by memory CD8 T cells can lead to rapid and effective control of influenza virus. Indeed, numerous studies have shown the benefits of immunological memory during heterosubtypic influenza virus infection [recently reviewed in detail in Ref. (3)]. Given the importance of memory CD8 T cell responses in influenza virus infection, it is important to understand the various factors that can affect the establishment and/or maintenance of immunological memory in the CD8 T cell compartment, particularly with regard to heterologous infections, which commonly occur in humans. Here, we review the effects of heterologous acute sequential or chronic co-infection on recruitment of the CD8 T cell response and memory generation and maintenance during influenza virus infection.

### CD8+ T Cell Immunity

T cells recognize peptides presented in the context of major histocompatibility complex (MHC) molecules located at the cell surface. The immunogenicity of a given epitope is dependent on many factors, including but not limited to: protein processing, protein affinity for MHC, frequency of epitope-specific T cells, and competition amongst other T cells for interactions with antigen presenting cells (APCs) (4). Immunogenic epitopes do not all stimulate the same magnitude of CD8 T cell response; rather, there is an immunodominance hierarchy, in which epitopes can be classified as dominant, codominant, or subdominant. Epitope immunodominance is not directly correlated with epitope abundance and appears at least partially dependent on the relative frequency of high avidity epitope-specific T cells, recruitment of CD8 T cell precursors, and the extent of precursor proliferation throughout the primary response (5, 6). In addition, an epitope’s immunodominance can change upon secondary infection. For example, during primary infection with the H3N2 lab strain X31, NP_366–374_/D^b^ and PA_224–233_/D^b^ epitopes elicit a CD8 T cell response similar in size; however, upon secondary infection with the H1N1 lab strain PR8, NP-specific CD8 T cells become dominant (7). The observed change in immunodominance upon secondary infection is associated with increased epitope presentation of NP (presented by multiple APCs) vs. PA [presented by dendritic cells (DCs) only], which augments activation and expansion of NP-specific memory CD8 T cells.

The CD8 T cell receptor (TCR) is composed of an alpha and beta chain. Each chain is generated by a semi-random recombination mechanism known as V(D)J recombination. In humans, the alpha locus consists of 42 variables (V) and 61 joining (J) segments; the beta locus consists of 47 V, 2 diversity (D), and 13 J segments. Diversity in the TCR is a result of three factors: (1) Semi-random pairing of a single V, D, and J segment. (2) Recombining of Vα-Jα or Vβ-Dβ-Jβ results in random nucleotide insertions and deletions at junction sites. (3) Combinatorial diversity of an alpha chain and a beta chain. The TCR generation process has the potential to generate 10^15^–10^61^ unique receptors (8–10). However, the size of the peripheral TCR repertoire in humans is estimated at ~10^6^–10^8^, and with many sequences overlapping between individuals, despite the enormous potential repertoire diversity (11–14). This is due, at least in part, to the preferential use of particular VDJ segments and positive-negative selection of T cells in the thymus, before entry into the periphery. The pool of T cells capable of recognizing a specific epitope is referred to as the epitope-specific T cell repertoire and comprised of various unique TCRs. On average, the size of an epitope-specific repertoire consists of 50–500 naive T cells (6, 15, 16).

Three signals contribute to the priming of a CD8 T cell: (1) Recognition of cognate antigen *via* interaction of TCR:peptide:MHC. (2) Interaction with activating co-stimulatory molecules. (3) Cytokines in the surrounding microenvironment. If the accumulation of these signals exceeds the threshold of activation, a T cell will be recruited into the T cell response and begin to proliferate. The T cell response occurs in three general phases: activation and expansion, contraction, and memory. Following activation, T cells undergo extensive division, replicating every 6–8 h and expanding up to 10^4^–10^5^ fold (17). Differentiation of CD8 T cells involves acquisition of effector functions, such as production of anti-viral IFN-γ, pro-survival IL-2, and cytolytic enzymes. Generally, the contraction phase begins following control of pathogen growth, during which 90–95% of activated T cells die *via* apoptosis by 2–3 weeks post peak expansion (17). The remaining CD8 T cells will further differentiate into various memory populations. There are three broad types of memory CD8 T cells commonly recognized: central memory T cells, T_CM_ (CD44^hi^ CD62L^+^ CCR7^+^ CD127^+^ CD69^−^ CD103^−^), circulate through secondary lymphoid tissues *via* the blood and lymph. Effector memory T cells, T_EM_ (CD44^hi^ CD62L^−^ CCR7^−^ CD127^+^ CD69^−^ CD103^−^), migrate throughout the periphery. Resident memory T cells, T_RM_ (CD44^hi^ CD62L^−^ CCR7^−^ CD11a^+^ CD69^+^ CD103^+^), remain in tissues and do not recirculate *via* the bloodstream. Memory CD8 T cells undergo epigenetic modifications that lead to a transcriptionally poised state, conferring rapid recall of effector function upon reencounter of a pathogen (18).

Given the high rate of mutations in influenza virus and potential for evasion of population immunity, it is imperative to understand how to optimize memory CD8 T cell responses, especially in the face of a new influenza subtype, during which CTL responses against conserved epitopes could play a key role in controlling infection. Most studies to date are conducted in specific pathogen free mice, in controlled environments, and do not take into account repetitive influenza infection throughout a lifetime, sequential acute heterologous infection between influenza infections, or co-infection with chronic heterologous infections. This is particularly important because humans may encounter numerous heterologous acute infections between influenza infections and the average adult is estimated to harbor ~8–12 chronic infections (19). Indeed, recent work has shown that mice infected with sequential heterologous infections, both acute and chronic, have immune responses to vaccination that are more human-like as compared with naive, specific pathogen free mice (20). Furthermore, in a study of influenza vaccine responses in humans, young CMV+ subjects had higher antibody titers and a generally activated immune system compared with young CMV-subjects (21). These data suggest infection history plays a role in shaping our response to immune challenge and may, at least in part, provide insight into the discrepancy between vaccination efficacies in the laboratory vs. in the clinic.

There are two general categories of heterologous infections—acute and chronic. It is important to note that in addition to acute infections, there are three distinct types of chronic infection that are often referred to interchangeably, but actually represent different scenarios for the immune system and conclusions from one category cannot be generally applied to another (Table 1). For this review, we will use the following definitions: (1) Acute, such as influenza virus infection, wherein T cells are transiently exposed to viral antigen and the virus is eventually cleared from the host (22–24). (2) Latent chronic, such as Epstein–Barr virus (EBV), where there are periodic phases of latency (no viral replication) and reactivation (production of infectious virus), during which T cells rest is exposed to antigen, respectively (25–27). (3) Smoldering chronic, such as Cytomegalovirus, wherein there is ongoing subclinical, low-level viral replication and T cells are continually exposed to antigen, with little rest (27–29). (4) Persistent chronic, such as Hepatitis C virus, where there is a continuous high-level of viral replication (viremia) and thus constant T cell stimulation with no periods of rest (30–34). In this review, when appropriate, sections will be divided into “Acute, Sequential” and “Chronic Co-infection.”

**Table 1:** Types of heterologous infections [modified from Ref. (17)].

Infection type	Category	Example	Characteristics	Antigen burden	Reference
Acute	–	Influenza virus	Eventual clearance of pathogen and transient exposure of T cells to antigen	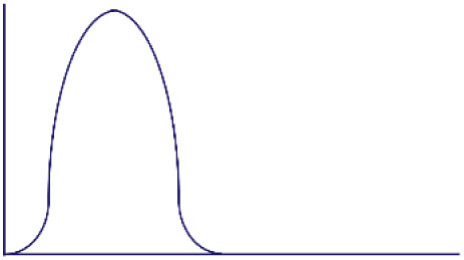	(22–24)

Chronic	Latent	Epstein–Barr virus and Herpes simplex virus	Chronic infection with periodic reactivation and periods of T cell exposure and rest	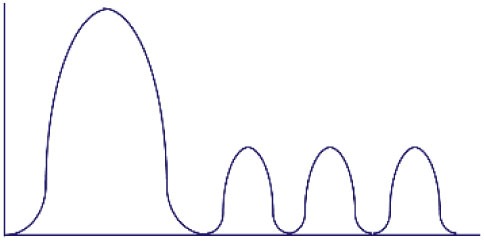	(25–27)

	Smoldering	Cytomegalovirus	Chronic infection with low-level ongoing viral replication and infrequent T cell rest	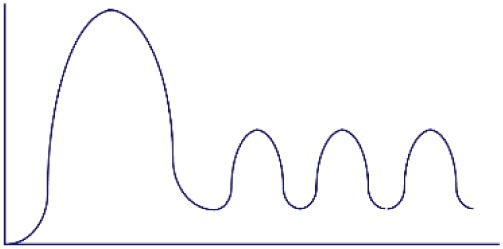	(27–29)

	Persistent	Hepatitis C virus and HIV/AIDs	Chronic infection with high viral replication (viremia) and no T cell rest, constant exposure to high levels of antigen	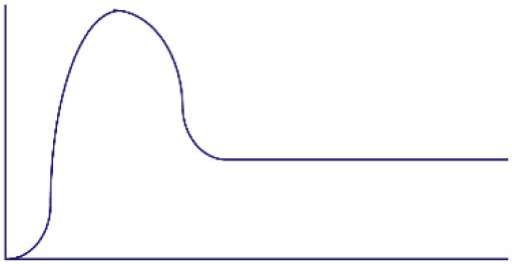	(30–34)

## Early Kinetics and Bystander Activation

Most studies are conducted in naive, specific pathogen free mice; however, humans encounter daily immune challenges that may impact pre-existing immunological memory and control of subsequent infections (homologous or heterologous). Indeed, prior infection with influenza virus protects mice from respiratory syncytial virus (RSV) induced eosinophilia and weight loss (35). Protection can be conferred *via* transfer of splenocytes from influenza virus-exposed animals, and is thought to be mediated by non-specific bystander activation, cross-reactive T cells, immunological imprinting (and skewing toward Th1 response), and/or structural remodeling after the first infection (35). This study highlights the impact infection history may have on the control and disease severity of a sequential heterologous infection.

Memory CD8 T cells are transcriptionally poised for rapid recall of effector function upon reencounter of a pathogen, and are capable of responding to 1/50th of the peptide concentration necessary for naive T cell stimulation (18, 36). Cytokine stimulation alone is even sufficient to induce memory CD8 T cell activation and cytokine production. Activation of T cells in the presence of an inflammatory microenvironment, but in the absence of cognate antigen, is termed bystander activation. Studies have shown bystander T cell proliferation can be induced by viruses, type I IFN, cytokines, and polyI:C (37, 38). Cytokines, including but not limited to, IFNα/β, IFN-γ, IL-12, IL-15, IL-7, and IL-18 have been shown to have unique and synergistic effects on bystander activation of T cells (39, 40). Furthermore, the extent of bystander activation depends on the infection dose and subsequent level of inflammation (41). Recent work in a primary influenza infection model shows early expansion of highly activated non-specific (bystander) memory CD8 T cells, which are CD25 negative, a component of the IL-2 receptor and a molecule that is up-regulated upon TCR stimulation, but are NKG2D positive, an activating receptor expressed on NK- and T-cells, and are restricted to the site of infection (42). Importantly, blockade of NKG2D resulted in increased influenza viral titers, suggesting a role for NKG2D in viral control (42). Similar results have been observed in a mouse model of *Listeria monocytogenes* (LM) infection, where bystander-activated memory CD8 T cells mediated early clearance of infection, in a NKG2D-dependent manner (43). Additional studies have shown NKG2D can act as a co-stimulatory molecule for CD8 T cells, augment cytotoxicity, and is sufficient to rescue unhelped memory CD8 T cells (44, 45). These results suggest a role for bystander-activated memory CD8 T cells in initial pathogen control.

### Acute, Sequential

An acute, sequential heterologous infection model of primary Sendai virus and secondary influenza virus infection, and the reverse sequence of infection, show early recruitment of non-specific memory CD8 T cells into the lung (46). Specifically, there is a 4–5 fold increase in Sendai virus specific CD8 T cells at day 4 after secondary influenza infection and a fourfold increase in influenza-specific CD8 T cells at day 4 after secondary Sendai virus infection. Similar results were observed in other respiratory infection models—there is 4.4 and 1.7 fold increase in murine herpesvirus 68 (MHV68)—and Vaccinia virus (VV)-specific CD8 T cells, respectively, at day 3 after secondary Sendai virus infection (46). Furthermore, in the primary Sendai and secondary influenza virus infection model, bystander Sendai-specific CD8 T cells exhibit a transiently altered phenotype at day 3–4 (coinciding with the peak of their presence in the lung), are recruited *via* circulating memory CD8 T cells, and recruitment is independent of proliferation, although a portion of non-specific cells do proliferate (46).

### Chronic Co-Infection

Epstein–Barr virus is a member of the gamma-herpesvirus family, and its seroprevalence in humans approaches 80–90% in adults (19). Studies using MHV68, a natural mouse pathogen that is closely related to human gamma herpesviruses, suggest latent MHV68 co-infection confers protection during challenge with influenza virus (47). Co-infected mice show enhanced survival, enhanced viral clearance at early time points, decreased lung injury, increased recruitment of activated CD4 and CD8 T cells at early and later time points, enhanced activation of alveolar macrophages, and augmented levels of anti-viral IFN-γ in response to influenza virus infection (47). Similar results were observed in a co-infection model of murine cytomegalovirus (MCMV) and influenza virus, wherein decreased influenza virus titers and increased numbers of influenza-specific CD8 T cells were observed in early (5 weeks) and established (12 weeks), but not long-standing (9 months), MCMV latently infected mice (21). Human cytomegalovirus (HCMV), a beta-herpesvirus family member, is also a significant human pathogen that infects approximately 50% of adults, with seroprevalence increasing up to 90% with age (48, 49). HCMV and EBV infections are largely subclinical and well tolerated; however, they are associated with significant increases in memory CD8 T cells over time, termed memory inflation. A study sought to determine whether unrelated virus specific memory CD8 T cells were activated in the immune response to acute, heterologous infections in humans (50). In peripheral blood mononuclear cells from patients at the onset of acute hepatitis B virus (HBV) infection, antigen-specific CD8 T cells within the total activated (CD38+ HLA-DR+) CD8 T cell compartment ranged (when detectable) from: 43 to 89% for HBV, 5.5 to 20% for HCMV, 22 to 41% for EBV, but only 0 to 2% for IAV (influenza A virus), as determined by pentamer binding. In addition, 54, 4.9, 8, and 0% of HBV-, HCMV-, EBV-, and IAV-specific CD8 T cells were proliferating, respectively. These results suggest that CD8 T cells specific for chronic pathogens may be preferentially activated during acute, heterologous infections. Moreover, acute infection with dengue, adenovirus, and influenza virus also induced activation of HCMV- and/or EBV-specific CD8 T cells (50). In one influenza case, approximately 25% of the onset activated CD8 T cells were HCMV-specific, but influenza-specific CD8 T cells could not be detected until day 5 (50). The study also found IL-15, a cytokine important for maintenance of memory T cells and often produced during acute viral infection, selectively activates HCMV- and EBV-specific, but not influenza-specific, CD8 T cells, is sufficient for spontaneous IFN-γ production, and enhances anti-viral cytokine production in conjunction with TCR stimulation (50).

These studies demonstrate that early recruitment of non-specific CD8 T cells is a common feature of respiratory infections, including influenza virus infection; however, analysis of viral load and illness outcome measures were not always included, and additional studies are needed to determine the extent to which bystander-activated CD8 T cells can contribute to the early immune response and control of a pathogen and/or the risk of immunopathology due to excessive T cell responses. It is likely that these effects are specific to the infection model studied.

## Cross-Reactivity and the T Cell Repertoire

It is estimated that a single TCR can recognize up to 10^6^–10^7^ foreign nonamer peptides and ≥10^8^ 11-mers (51). Degeneracy of the TCR repertoire facilitates heterologous immunity *via* cross-reactive T cells primed during primary infection and activated during a secondary, unrelated infection. This may be advantageous in defense against influenza infection where T cells primed from a previously circulating influenza virus strain may respond to a novel, antigenically distinct strain.

### Acute, Sequential

With respect to influenza virus infection, there are two types of acute, sequential heterologous infection scenarios. First, heterosubtypic influenza immunity refers to the effect of pre-existing immunity to influenza virus strain 1 on the immune response to a secondary infection with influenza virus strain 2. Studies in mice show priming with H9N2 or H1N1 confers protection against challenge with H7N9; however, CD8 T cell immunodominance hierarchies, weight loss, and viral clearance varied by the priming influenza virus strain (52). Although the diversity of the CD8 TCR repertoire and presence of cross-reactive CD8 T cells was not specifically tested, this study highlights the significance infection history may have on cell mediated immunity and illness outcome.

The second scenario is the effect of pre-existing immunity against an acute, non-influenza virus infection on the quality or magnitude of the immune response to influenza virus infection, and *vice versa* (discussed more in the Section “Maintenance of Memory and Attrition”). The effects of heterologous infections on influenza immunity (either establishment or maintenance of memory) have not been sufficiently studied; however, studies with acute lymphocytic choriomeningitis virus (LCMV), VV, and Pichinde virus (PV) infections in the mouse model show that heterologous infection can induce activation of putatively cross-reactive CD8 T cells and alter LCMV-specific T cell immunodominance (53). However, the sequence of infection is important and heterologous protective immunity is not necessarily reciprocal, i.e., LCMV confers protection against VV, but VV does not protect against LCMV (53). Additional work in the acute LCMV–PV model shows PV-immune mice infected with LCMV exhibit an altered immunodominance hierarchy, such that the immunodominant epitope is NP_205_, a normally subdominant epitope with high sequence similarity between the two viruses (six out of eight amino acids) (54). Alterations in immunodominance and the CD8 TCR repertoire may change the pool of memory CD8 T cells, and thus could impact secondary immune responses. Given that humans may encounter various infections between influenza infections, it is imperative to understand how heterologous infections may alter influenza-specific immunity and subsequently illness outcome.

### Chronic Co-Infection

**Figure 1:**
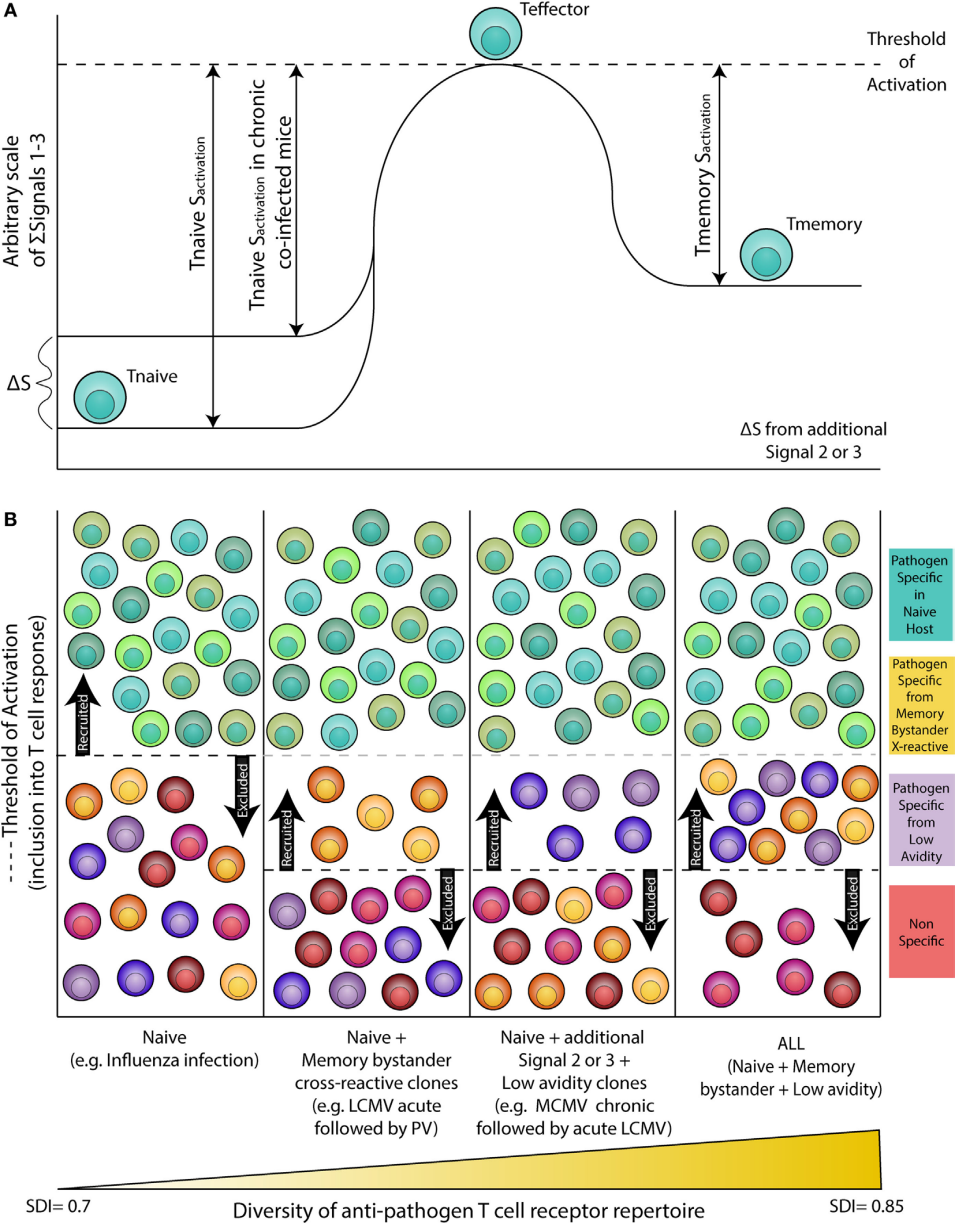
Altered threshold of CD8 T cell activation during latent and/or smoldering chronic co-infection or from memory bystander activation. **(A)** Three signals contribute to the activation and recruitment of naive CD8 T cells into a specific anti-pathogen response: (1) the T cell receptor (TCR), (2) co-stimulation, and (3) cytokines in the microenvironment. If the sum of these signals exceeds the threshold of activation (Tnaive s_activation_), the naive CD8 T cell will be recruited into the immune response. Chronic co-infection is known to augment (ΔS) basal cytokine levels and activation status of antigen presenting cells, thereby decreasing the signal required by naive CD8 T cells to exceed the threshold of activation (Tnaive s_activation_ in chronic co-infected mice). In addition, memory CD8 T cells are transcriptionally poised for rapid recall of effector function upon reencounter of a pathogen, thus the amount of signal required for inclusion of a memory cell (Tmemory s_activation_) is smaller than that of a naive T cell. **(B)** Most infectious disease studies are conducted in a naive host (far left panel), in which challenge with a pathogen will result in recruitment of pathogen-specific CD8 T cells from the naive T cell pool (blue-green T cells). In previously infected hosts, memory T cells have a lower threshold of activation and can contribute unique cross-reactive clones (yellow-orange T cells, center left panel). In addition, chronic co-infected hosts have altered signals 2 and 3 that permit the recruitment of lower avidity clones (purple T cells, center right panel). Addition of memory bystander and lower avidity clones increases the diversity of the anti-pathogen CD8 TCR repertoire. Most humans have at least one chronic infection and encounter multiple infections throughout a lifetime, thus a combination of all three scenarios (far right panel) more accurately depicts what occurs in life, and represents the most diverse population of pathogen-specific CD8 T cells.

Altered activation of APCs (signal 2) and cytokine levels (signal 3) as a result of chronic co-infection may decrease the threshold of activation for inclusion into the T cell response, allowing for recruitment of lower avidity and/or cross-reactive T cells that may otherwise not be included in the response (Figure 1). Indeed, studies in humans show the naive T cell pool in chronic hepatitis C virus (cHCV)-infected subjects has more biased Vβ segment usage and decreased expression of CD5, a known T cell co-inhibitory receptor (55). Upon low dose anti-CD3 and anti-CD28 stimulation, compared with healthy donors, naive T cells from cHCV patients showed increased ERK phosphorylation, higher frequency of CD25 and CD69 expression, and activation induced cell death (55). In addition, work by Che et al. shows acute LCMV immune mice infected with MCMV exhibit increased MCMV viral titers and enhanced immunopathology. Conversely, prior MCMV infection conferred protection against acute LCMV infection *via* augmented CD8 T cell responses against a normally subdominant LCMV epitope, L_2062–2069_, mediated *via* cross-reactivity with a MCMV epitope, M57_727–734_ (56). Furthermore, studies show chronic infection with LCMV (IFN inducing), *Toxoplasma gondii* (*T. gondii*, IL-12 inducing), or *Heligmosomoides polygyrus* (*H. polygyrus*, Th2 inducing) lead to impaired development of immune memory and protective immunity (57). This study also found that there is a distinct transcriptional profile between HCMV-specific (A02*01 pp65_495_, NLVPMVATV and B07*02 pp65_417_, TPRVTGGGAM) memory CD8 T cells from healthy vs. persistent HCV infected humans (57). Gene set enrichment analysis shows the gene expression profile of HCMV-specific memory CD8 T cells in healthy donors and also found enriched in memory OT-I cells from naive mice, but not in chronic LCMV-infected mice (57). Collectively, these data demonstrate that chronic infections may: (1) Alter the basal status of naive CD8 T cells, such that they are hyperactivated upon stimulation. (2) Enhance CD8 T cell responses *via* inclusion of cross-reactive clones. (3) Alter the transcriptional profile of memory CD8 T cells. These results underscore the importance of studies which explore the extent to which this impacts influenza cell mediated immunity, whether the results are collectively beneficial or detrimental to the host, and to what degree the results depend on the infection model and/or sequence of infection.

A study of HCV-specific T cell responses confirmed cross-reactivity between epitopes from two unrelated viruses can occur in humans. Specifically, approximately 60% of HCV negative, healthy controls have functionally cytotoxic memory (CD45RO+) CD8 T cells specific for an immunodominant HCV epitope, A02*01 NS3_1073_ (CVNGVCWTV) (58). The NS3_1073_ epitope shares seven of the nine amino acids with the influenza virus A02*01 NA_231_ epitope (CVNGSCFTV), with conserved residues at positions key in binding to HLA A02*01. Indeed, HCV negative controls with NS3-specific CD8 T cells showed functional responses to NA_231_ and A02*01 M1_58_ (a known immunodominant influenza epitope), suggesting prior influenza virus exposure (58). Furthermore, NS3-specific T cells could be induced by influenza infection in A02*01 transduced mice (58). Although the effects on control of influenza infection are not clear, it is possible that HCV infection will lead to a more narrow anti-influenza T cell response, due to expansion of HCV-specific CD8 T cells (both non cross-reactive and cross-reactive) in an attempt to limit HCV replication, and will ultimately result in poor influenza illness outcome.

Cross-reactive T cells against an influenza and heterologous virus epitope were also observed in EBV-associated infectious mononucleosis (IM) patients, where two out of eight patients had tetramer-defined cross-reactive CD8 T cells specific for EBV A02*01 BMLF_280–288_ (GLCTLVAML) and IAV A02*01 M1_58–66_ (GILGFVFTL), despite only 33% sequence homology (59). This result is further supported by sequencing of the CDR3β regions of Vβ17+ cells (from M1-specific cell lines), which shows that diversity of Jβ segment usage in the influenza M1-specific Vβ17+ TCR repertoire changed throughout IM disease progression (59). Furthermore, follow up studies show while cross-reactive and non cross-reactive BMLF-specific T cells utilize the Vβ14 segment, sequencing of the CDR3β loop of the cross-reactive clones showed 64% of them were not previously observed in the non cross-reactive repertoires (60). Analysis of TCR-α chain segment usage shows cross-reactive and non cross-reactive repertoires utilize Vα15, but cross-reactive repertoires utilize unique Jα families (60). In addition, BMLF-M1 cross-reactive T cells utilize a greater number of Vβ segments, as compared with non cross-reactive M1 or BMLF-specific T cells (60). These results have two important implications: (1) Cross-reactive T cells can increase TCR repertoire diversity through inclusion of unique TCRs that would not be seen in single epitope-specific repertoires. (2) Compared with analysis of segment usage alone, analysis of CDR regions provides more insight to the number of unique clones and diversity of the TCR repertoire. It is important to note that segments of interest when comparing usage between cross-reactive and non cross-reactive repertoires were dependent on the individual, with some segments common across individuals (possibly reflective of “public” clones), while others were unique to individuals (possibly reflective of “private” clones) (60). This study also utilized computer simulations of cross-reactive responses and the results suggest that cross-reactive responses between structurally similar epitopes, termed “near cross-reactive” responses, will lead to a more narrow TCR repertoire, whereas cross-reactive responses between structurally divergent epitopes, termed “far cross-reactive” responses, would lead to a broad TCR repertoire (60).

### Reciprocal Effects of Influenza Infection

Numerous studies have shown that heterologous infections can impact influenza immunity and/or illness outcome, but the effect of influenza infection on the control of heterologous infection has not been sufficiently studied, and the extent to which these scenarios are dependent on cell mediated immunity is not clear. We previously mentioned prior infection with influenza virus protects mice from RSV induced eosinophilia and weight loss, and is thought to be mediated by non-specific bystander activation, cross-reactive T cells, immunological imprinting (and skewing toward Th1 response), and/or structural remodeling after the influenza infection (35). However, the aforementioned IM study observed BMLF-M1 cross-reactive T cells were enriched in severe IM cases, suggesting the magnitude of the anti-EBV CD8 T cell response is associated with disease severity (59). Indeed, later work in a study of acute infectious mononucleosis (AIM) shows IAV-M1 (R^2^ = 0.4), EBV-BMLF (R^2^ = 0.3), and cross-reactive IAV-M1 + EBV-BMLF (R^2^ = 0.6) CD8 T cells are the only tetramer positive populations which directly correlate with AIM disease severity and are predictive of severe AIM in a relative-risk analysis (61). Other tetramer positive populations analyzed include CMV-pp65, EBV-BLRF1, cross-reactive IAV-M1 + EBV-BLRF1, and cross-reactive EBV-BLRF1 + EBV-BMLF. These results suggest influenza infection history and the frequency of influenza-EBV cross-reactive CD8 T cells in the influenza memory T cell pool may alter anti-EBV cell mediated immune responses during acute infection and subsequent illness outcome (61).

Taken together, these data demonstrate heterosubtypic influenza infections, acute sequential heterologous infections and chronic co-infections can alter anti-influenza memory CD8 T cell responses, with respect to kinetics, magnitude, quality, and repertoire diversity. Alterations in signals 2–3 of T cell priming may alter the threshold of activation and subsequently the pool of CD8 T cells included in the anti-influenza response. If this leads to a more diverse TCR repertoire, it could be beneficial in the face of a novel influenza virus strain; conversely, if it results in a narrow repertoire, it may lead to variant escape. Further studies are needed to assess the consequences of common heterologous infections on influenza virus immune memory; does cross-reactivity narrow or diversify the TCR repertoire, and to what extent are the effects context/infection dependent? In addition, what are the reciprocal effects of influenza infection on control of heterologous infections?

## Maintenance of Memory and Attrition

It is well established that the ability to mount an effective immune response declines with age, and annual surveillance of influenza infections show the elderly (>65 years old) are at risk for severe disease from influenza infection (62, 63). Immunosenescence is associated with poor immune responses and is a collective term used to describe various changes in the immune system that occur over time, such as thymic involution, dysregulated innate immune responses, inverted CD4/CD8 T cell ratios, decreased naive T cells coupled with increased memory T cells, and decreased diversity in the TCR repertoire (64–67). Alterations in naive/memory T cell frequencies, often observed in the elderly, are partially due to exposure to numerous pathogens throughout a lifetime, decreased thymic output, and the large expansion of CMV-specific CD8 T cells, termed “memory inflation”(68–70).

Altered immunological memory as a result of pathogen exposure throughout a lifetime coupled with memory inflation from chronic infections including CMV raise the concern that heterologous infections could consume the limited space in the memory compartment, “crowding out” protective memory responses to influenza virus. However, recent studies show the CD8 T cell compartment can grow in size upon immunological experience (71). In this study, mice were infected with LCMV, followed by three heterologous prime-boost vaccines against vesicular stomatitis virus: New Jersey strain, recombinant VV expressing VSV nucleoprotein, and Indiana strain. The memory CD8 T cell population specific for the N protein of VSV induced by this vaccination strategy was equivalent in size to the entire memory CD8 T cell population in control mice (71). Furthermore, the total number of CD8 T cells increased following sequential vaccination, highlighting the importance of analyzing and reporting both cell number and frequency, as a decreased frequency may be the result of increased cell numbers of other CD8 T cells (71). Importantly, moderate attrition occurred in non cross-reactive, P14 LCMV-specific CD8 T cells in various tissues, ranging from 25.6 up to 33.4%. However, no attrition was observed in LCMV-specific CD8 T cells when mice were sequentially infected with VV, LM (intracellular bacteria), or *Plasmodium yoelii* (parasite) (71). These results suggest the CD8 T cell compartment size is flexible, though this flexibility appears to depend on specific features of the infecting pathogens, such as induced innate immune profiles, that we do not currently fully understand; however, with additional investigation and careful vaccine design, the magnitude of memory CD8 T cell attrition may be reduced following repeated heterologous challenge.

Two models have been suggested for memory T cell attrition: (1) Passive competition, in which new memory T cells compete with pre-existing memory T cells for space in limited survival niches and (2) Active deletion, wherein some mechanism, such as early type I IFN, induces apoptosis of pre-existing memory T cells, to make room for newly arising memory T cells (72). Attrition of T cells during the early phase of an acute immune response is mediated, at least in part, through type I IFN (IFN-α/β), followed by activation of initiator caspase 8 and effector caspase 3, ultimately leading to apoptosis (73, 74). Additional studies in mice show loss of T cells during early infection is age dependent, such that aged mice are less susceptible to T cell attrition mediated by type I IFN due to decreased expression of caspase 3, as compared with young mice (75). This is significant because thymic output decreases as age increases; a lower apoptotic potential of memory T cells in an aged host would minimize loss of this population when limited naive cells are available to replace them.

LCMV, PV, VV, and MCMV studies in mice demonstrate heterologous infections have prospective and/or retrospective effects on immune responses and memory: prospectively, prior infection with Virus A can lead to beneficial or detrimental effects during sequential infection with Virus B, and reciprocal (Virus B → Virus A) effects are not necessarily equal (53, 76). Retrospectively, infection with Virus B in a Virus A-immune host leads to the loss of bystander-activated T cells, including Virus A-specific memory T cells (74, 76–78). Studies of influenza and MHV68 show reciprocal effects in a challenge and vaccination model (79). Compared with an influenza only control group, mice infected with influenza virus (PR8 then X31) followed by MHV68 exhibit decreased frequencies and numbers of influenza (NP) specific memory CD8 T cells in the spleen, peripheral blood, lung, and bone marrow; however, no difference was observed in the mediastinal lymph node (MLN), cervical lymph node (CLN), or liver at day 100 (79). Compared with MHV68 only mice, mice infected with influenza followed by MHV68 showed decreased numbers of total MHV68 (p79)-specific memory T cells (sum of all anatomical locations tested); however, this is likely driven by a difference in the liver at day 100, because no difference was observed in the spleen, peripheral blood, MLN, CLN, bronchoalveolar lavage, lung, or bone marrow (79). Moreover, vaccination for MHV68 followed by influenza infection resulted in a higher number of influenza-specific CD8 T cells at day 14, but a lower number at day 200 (79). In each scenario, the reduction of antigen-specific T cells was approximately twofold or less, and likely would not result in a loss of protection following secondary challenge with influenza virus or challenge post priming for MHV68 (79). Latent MHV68 infection actually confers protection against influenza infection (described in more detail in the Section “Early Kinetics and Bystander Activation”) (47).

These studies utilized MHV68, a murine model for chronic EBV infection in humans; however, most attrition studies are conducted in acute, sequential infection models. Given the high prevalence of chronic infections in humans, it is important to consider how they may alter observations of T cell attrition, and how this may vary by the category of chronic infection (latent, smoldering, or persistent). A study of PV, LCMV strain Armstrong (acute), and LCMV clone 13 (persistent chronic) sought to examine these differences and found more profound attrition of PV-specific memory T cells in chronic (clone 13) sequential vs. acute (Armstrong) sequential infection (80). Importantly, CD44^hi^ memory CD8 T cells and non cross-reactive T cells were more susceptible to attrition (80). One possible explanation for the differences observed between acute and chronic LCMV, and important factors to consider when comparing chronic infection models, is the duration of antigen burden and the magnitude of subsequently induced pro-inflammatory cytokines. Studies in mouse models show out-of-sequence signal 3, such as the strong cytokine stimulatory conditions induced by sepsis and systemic immunotherapy, can lead to transient immunosuppression of T cells which is mediated, at least in part, through increased expression of suppressor of cytokine signaling, likely as a means to prevent extensive immunopathology from hyperactivation or autoimmunity (81, 82). For influenza infection, detrimental effects are likely to arise from persistent chronic co-infection, such as chronic LCMV; whereas smoldering MCMV- and latent MHV68-influenza co-infection models have shown enhanced CD8 T cell responses and improved illness outcome (21, 47).

Earlier studies have also suggested that memory T cells are maintained through cross-reactive stimulation, and recent work further supports this hypothesis (83, 84). In a mouse model of LM (wild type or recombinant expressing OVA) followed by *Mycobacterium bovis* (BCG, wild type or recombinant expressing OVA), mice infected with LM then BCG showed significant reduction in LM-specific CD4+ and CD8+ T cells; however, attrition did not occur in mice infected with LM-OVA followed by BCG-OVA (85). These data show heterologous bacterial, sequential infections also lead to T cell attrition, but attrition can be prevented when T cells cross-react across pathogens (85). Utilizing PV and LCMV acute, sequential infection models, Brehm et al. (discussed in more detail in the Section “Cross-Reactivity and the T Cell Repertoire”) has also demonstrated that cross-reactive NP_205_-specific T cells are preferentially maintained at a higher frequency as compared with non cross-reactive T cells, whether LCMV is given to PV-immune mice or PV is given to LCMV-immune mice (54).

These data have shown that attrition of memory T cells is a common phenomenon in a variety of viral and bacterial infection models. In addition, non cross-reactive clones are more susceptible to attrition, whereas cross-reactive clones are maintained. Thus, it is possible that analysis utilizing known immunodominant epitopes may not account for increases in frequency and number of cross-reactive T cells specific for normally subdominant responses not analyzed. To provide evidence for this possibility, more studies are needed across a broader range of specificities, such as all activated CD8 T cells rather than just tetramer-specific cells, coupled with a more detailed analysis of paired αβ TCR sequences to look for the expansion of cross-reactive clones. In-depth analysis of TCR sequences can address additional questions, such as how different is the TCR repertoire pre and post attrition? Does attrition result in fewer numbers of each clone or complete loss of specific clones? If the latter, is there a selective mechanism, such as T cell phenotype (T_CM_ vs. T_EM_), perhaps with varying transcription of genes involved in the apoptotic process, or divergent vs. canonical TCRs (with respect to epitope and pathogen)? In addition, to what extent do observations depend on features of the infection model, such as a restricted site of infection vs. systemic, Th1 vs. Th2 bias, low vs. high pathogenicity, and the extent to which CD8 T cells contribute to pathogen clearance? The answers to each of these questions have important implications for control of influenza virus infection and the development of prophylactic methods. For example, if attrition preferentially results in the loss of divergent TCRs or complete loss of specific clones, this may lead to influenza virus escape variants due to decreased diversity in the influenza-specific CD8 TCR repertoire. Understanding the factors that affect memory T cell attrition can be utilized in the development of more effective influenza vaccines that minimize the loss of pre-existing memory CD8 T cell populations, such as choosing adjuvants which limit type I IFN production or skew Th1/Th2 ratios to preferential levels.

## Resident Memory

Resident memory T cells (T_RM_) reside in nonlymphoid tissues and serve as the first line of defense upon secondary infection. Histological examination of uninflamed human lung, counting CD3 positive cells in the lung parenchyma, suggests there are approximately 1 × 10^10^ resident T cells (86). Human influenza-specific lung CD8+ T_RM_ cells exhibit high proliferative capacity, are polyfunctional, and have a diverse paired TCR repertoire, likely a key attribute to prevent viral escape variants (87). Indeed, a comparison of human CD8+ T_EM_ in the blood and lung CD8+ ${\text{T}}_{{\text{RM}}^{{\;}^{\text{CD103+}}}}$ cells show distinct chemokine and adhesion molecule profiles, reflective of their corresponding localization (88). For example, lung ${\text{T}}_{{\text{RM}}^{{\;}^{\text{CD103+}}}}$ cells were enriched for CXCR3, CXCR6, and CCR5, but expressed low levels of CX3CR1, a chemokine receptor that mediates migration from circulation. Without *in vitro* stimulation, lung ${\text{T}}_{{\text{RM}}^{{\;}^{\text{CD103+}}}}$ cells also expressed higher mRNA, but lower protein, levels of effector molecules, such as granzyme B, IFN-γ, and TNF (88). Conversely, blood T_EM_ had higher granzyme B levels, despite lower mRNA levels. Lung ${\text{T}}_{{\text{RM}}^{{\;}^{\text{CD103+}}}}$ cells also expressed higher mRNA levels of chemokines and inhibitory molecules at a resting state, and expressed higher levels of IFN-γ upon stimulation with phorbol ester PMA and ionomycin (88). These results show lung T_RM_ are transcriptionally poised to mediate rapid effector responses and recruitment of additional leukocytes, while expression of co-inhibitory molecules may represent a means to prevent excessive immune responses and subsequent immunopathology (88).

Characteristic markers to identify T_RM_ cells in the epithelium include: (1) αE(CD103)β7 integrin, which interacts with E-cadherin in the epithelia and mediates retention in the lung and (2) C-type lectin CD69, an activation marker associated with recent antigen exposure, but also up-regulated in response to cytokines, such as type I IFN and TNF-α (89–91). It is important to note, not all T_RM_ express these markers and a comparison of T_RM_ at three anatomically distinct sites (lung, skin, gut) shows expression of 37 commonly up- or down-regulated genes, but 25–127 transcripts unique to T_RM_ from a given location (92, 93). An estimate of CD103+ CD8+ αβ T cells suggests this population comprises approximately 1/3 of the total T cells in the human lung and is primarily located above the basement membrane of small airways (94). Turner et al. has shown anti-influenza CD4+ and CD8+ T_RM_ cells localize to distinct niches in the lungs near airways and bronchovascular bundles, and were maintained independently of circulating and lymphoid T cell reservoirs (95). Analysis of human T_RM_ cells also shows compartmentalization depending on the site of viral infection; specifically, influenza-specific CD8 T cells were enriched in lung T_RM_ vs. the spleen, whereas similar frequencies of CMV-specific CD8 T cells were found in the lung and spleen (95). An independent study in humans also observed selective localization of antigen-specific CD8 T cells. The lungs were enriched for influenza-specific and RSV-specific CD8 T cells compared with blood, but CMV and EBV-specific CD8 T cells were equally distributed between both locations (96).

T_RM_ utilize a variety of methods to enhance the immune response and improve illness outcome, including, but not limited to: upregulation of adhesion molecules important for leukocyte migration, maturation of DCs, activation of NK cells, and rapid upregulation of broadly active anti-pathogen genes (97–100). In addition, lung T_RM_ have enhanced survival during influenza infection due to higher expression of anti-viral IFITM3, which confers protection against viral infection (101). Their location at the epithelial layer (which is the initial site of influenza virus infection) and rapid effector function make T_RM_ a key population in the initial control of influenza virus replication after secondary infection. Indeed, heterosubtypic influenza challenge models and studies of vaccines which induce lung CD8+ T_RM_ demonstrate the protective effects of this population during influenza infection; these results highlight the importance of understanding the various factors which may affect the generation and maintenance of T_RM_ (102–107). To date, studies in influenza infection models have demonstrated the importance of four general factors in the generation and maintenance of T_RM_: cytokines, co-stimulation, APC differentiation, and antigen (presence and avidity). Importantly, the extent to which a factor impacts T_RM_ may vary by anatomical location, and requires further investigation.

CD4+ T cells can enhance activation and differentiation of CD8+ T cells through cytokine production (Signal 3), and licensing of DCs, leading to enhanced expression of co-stimulatory molecules (e.g., 4-1BBL, Signal 2) which engage cognate receptors on CD8 T cells (e.g., 4-1BB). Indeed, studies of primary influenza infection in 4-1BB^+/+^ and 4-1BB^−/−^ mixed bone marrow chimeras show 4-1BB^−/−^ CD8+ T cells have an impaired ability to develop into lung T_RM_, indicating a role for this co-stimulatory pathway in the generation and/or maintenance of T_RM_ (108). Furthermore, studies in a CD4 depleted mouse model of influenza virus challenge show IFN-γ+ CD4+ T cells are important for the development of CD8+ T_RM_ (109). Unhelped CD8+ T cells have decreased T_RM_, impaired ability to confer heterosubtypic protection, and show enhanced Tbet expression (109). Overexpression of Tbet in CD8 T cells abrogates transforming growth factor-beta (TGF-β) induced expression of CD103, *via* binding at the first intron in the *Itgae* (CD103) locus and blocking a putative Smad3 binding site (109). Importantly, these observations may also reflect differences in DC licensing, as CD4+ T cell depleted mice will also lack CD4 T cell:DC interactions. Indeed, previous work in a HSV mouse model has shown that unhelped DCs exhibit decreased proliferation and expression of IL-2R, IL-7R, and anti-apoptotic Bcl-2 (110). Moreover, anti-HSV CD8 T cell responses in MHC II knockout and/or CD4 T cell antibody depleted mice were impaired at peak primary (day 7 post infection) and memory (90–110 days post priming) time points (110).

In addition to activation and co-stimulatory molecule expression, human and mouse models have suggested DC phenotype is an important factor in the activation and differentiation of CD8 T cells. Human blood DCs can be divided into three categories based on cell surface marker expression; CD303+ (also known as BDCA-2) expressed on plasmacytoid DCs (pDCs), CD1c (also known as BDCA-1) expressed on most circulatory DCs, and CD141 expressed on a smaller subset (111). Studies of influenza vaccine responses in human tissues and humanized mice show both human CD141+ CD1c- and CD1c+ DCs are capable of activating and inducing expansion of influenza-specific memory CD8+ T cells; however, CD1c+ DCs have an enhanced capability to induce differentiation of CD103+ CD8+ T cells which express effector molecules, such as granzyme B, and are retained in the epithelium (111). Expansion of CD103+ CD8+ T cells by CD1c+ DCs was TGF-β dependent—a cytokine with a well established role in the regulation of CD103 expression on memory T cells (91, 93, 111). Mouse challenge models of influenza have also shown a differential capacity of respiratory dendritic cell (RDC) subsets to activate CD8 T cells during influenza infection and further support the human results. Specifically, CD103+ RDCs (CD103+ MHCII^hi^ CD11b^neg-hi^) and CD11b^hi^ RDCs (CD103- MHCII^hi^ CD11b^med-hi^) exhibit higher antigen uptake, increased expression of co-stimulatory and antigen presenting molecules (CD1d and MHC II), and decreased expression of inhibitory molecules (B7-H1) as compared with moRDCs (CD103- MHCII^neg-med^ CD11b^hi^) and pDCs (B220+ Gr-1 + MHCII^lo^) (112). Furthermore, although CD103+ and CD11b^hi^ RDCs similarly activate CD4+ T cells in terms of proliferation and cytokine production, CD103+ DCs induced more robust activation of CD8 T cells, leading to increased proliferation and production of effector molecules (e.g., granzyme B and IFN-γ) (112). Additional studies support differential activation of CD8 T cells by CD103+ RDCs and CD11b^hi^ RDCs, as determined by homing/migration patterns, proliferation, and the expression of activation markers, effector molecules, and transcription factors important for T cell fate (113).

The respiratory tract can be divided into two sections; the upper respiratory tract (URT) is comprised of the nose, mouth, and pharynx, whereas the lower respiratory tract (LRT) includes the trachea, bronchi, and lungs. Studies by Pizzolla et al. show distinct requirements between T_RM_ in the upper vs. LRT. T_RM_ in the URT can develop independently of local cognate antigen and TGF-β, exhibit increased longevity over time, and are sufficient to prevent dissemination of influenza infection from the URT to the LRT, thereby protecting against severe disease (104). These results suggest the factors that affect the generation and/or maintenance of T_RM_ can vary by location. In addition, comparison of lung CD8+ T_RM_ against two immunodominant influenza epitopes, NP_366–374_/D^b^ and PA_224–233_/D^b^ shows distinct transcriptional profiles, suggesting a role for specific pMHC:TCR interaction parameters, such as avidity, in the differentiation of T_RM_, in addition to presence of cognate antigen (114).

After the resolution of infection, lung T_RM_ wane over time and this loss is associated with impaired control of heterosubtypic influenza virus challenge. Recent work by Slütter et al. shows loss of lung-resident T_RM_ is due to apoptosis rather than migration, and T_RM_ maintenance in the short term depends on immigration of circulating CD8+ memory T cells. In addition, T_EM_ are precursors of lung T_RM_, boosting of T_EM_ results in increased frequency of T_RM_ in lung, and TNF-α is important for recruitment and conversion of memory CD8 T cells to T_RM_ phenotype (115). Furthermore, late circulating memory CD8 T cells (>100 days post infection) have an inherently decreased capacity to form lung T_RM_, as compared with early circulating memory cells (20–30 days post infection); this is reflected by differences in transcriptional profiles (115). Compared with early memory, late memory CD8 T cells have differential expression of three T_RM_ master regulators (Eomes, Blimp-1, and Hobit) and decreased expression of various genes important for T cell migration (115).

Heterologous infections have been shown to alter epitope immunodominance, CD4+ T cell Th1/Th2 ratio, cytokine levels (e.g., TNF-α), and augment expression of antigen presentation and co-stimulatory molecule expression on APCs (35, 47, 54, 116). Collectively, these data suggest sequential acute and chronic co-infection may alter factors known to play a key role in the establishment and maintenance of T_RM_, and underscore the importance of studies which examine these relationships. Increased numbers or diversity of lung CD8+ T_RM_ could result in more rapid control of influenza virus and improved illness outcome, or excessive responses and immunopathology. However, vaccination studies suggest the former is more likely, and transcriptional analysis of T_RM_ suggests they have augmented expression of co-inhibitory molecules, which likely act to minimize immunopathology (88, 102, 104–107). Alternatively, decreased numbers or diversity of lung CD8+ T_RM_ may lead to variant escape, impaired control of influenza virus growth, and immunopathology due to prolonged inflammatory immune responses.

## Conclusion

Memory T cells specific for conserved influenza epitopes can be advantageous during an outbreak of an antigenically distinct viral strain. Indeed, several vaccine studies in mouse models show vaccines which boost memory CD8 T cells can confer protection during heterosubtypic challenge. However, while a robust memory CD8 T cell population can lead to a rapid immune response to secondary infection and protect against severe disease, detrimental effects are possible. Cross-reactive clones may dominate the response to heterologous challenge and lead to a narrowed TCR repertoire. As previously discussed, heterologous infection with LCMV and PV alters the immunodominance of the CD8 T cell response, such that a normally subdominant NP_205_ epitope becomes immunodominant (54). Additional work in this model shows the TCR repertoire is narrowed to the extent that it results in an escape variant (117). Furthermore, studies in various infectious disease models have demonstrated an excessively large CD8 T cell response may lead to enhanced immunopathology and more severe illness outcome (56, 61, 118, 119).

Collectively, the studies reviewed here demonstrate that various heterologous infection scenarios can alter the primary T cell response and establishment or maintenance of the memory CD8 T cell pool. Specifically, heterologous acute sequential and chronic co-infection may result in: early migration of CD8 T cells to the site of infection, altered immunodominance hierarchies, inclusion in the anti-viral response of cross-reactive and/or lower avidity CD8 T cells, changes in cytokine levels, altered transcriptional profiles of naive and memory T cells, and changes in the differentiation of APCs (Table 2). Augmented activation of APCs (i.e., higher expression of co-stimulatory molecules) and/or production of cytokines (by APCs or CD4s) important for CD8 T cell differentiation or bystander activation of memory CD8 T cells would lower the threshold of activation for inclusion into the anti-influenza T cell response, thereby increasing the diversity of the TCR repertoire by facilitating the inclusion of low avidity and/or cross-reactive clones that would otherwise not be present. Thus, additional studies which more accurately reflect pathogen encounters in humans are needed to optimize vaccination strategies by inducing diverse, local memory CD8 T cell responses, while minimizing loss due to attrition and preventing immunopathology due to excessive pro-inflammatory responses.

**Table 2:** Overview of studies showing heterologous acute sequential or chronic co-infection can alter influenza virus immunity.

Infection type	Species	Priming strain (vaccine or infection)	Secondary strain (vaccine or infection)	Experimental design	Disease outcome	Effects on magnitude or quality of immune response	Reference
Acute, sequential (heterosubtypic)	C57BL/6	Influenza virus (H9N2 and H1N1)	Influenza virus (H7N9)	Mice were primed with 10^4^ TCID_50_ of H9N2 or 10^2^ TCID_50_ of H1N1 intranasally, and challenged with H7N9 intranasally at 10–12 weeks post priming	Mice primed with H9N2 or H1N1 showed increased survival, enhanced viral clearance, and decreased weight loss compared with naive mice	Prior infection with H9N2 or H1N1 leads to early and robust CD8 T cell responses during secondary infection with an antigenically distinct influenza virus, H7N9. Importantly, the magnitude of the priming-virus memory CD8 T cells was the best correlate of protection against H7N9 challenge. In addition, the degree of conferred protection (i.e., viral clearance, weight loss profile, and survival) and immunodominance of CD8 T cell responses varied by the priming-virus strain.	(52)

Acute, sequential	BALB/c	Influenza virus (X31 and H3N2)	Respiratory syncytial virus (RSV, A2 strain); recombinant Vaccinia virus (VV) expressing RSV attachment protein (rVV-G) or control β-galactosidase (rVV-β-gal)	Mice were infected with 3 × 10^6^ PFU human RSV, 50 hemagglutinin (HA) units of X31, or HEp-2 lysate intranasally at day 0. Three to five weeks later, they were infected with 3 × 10^6^ PFU rVV-G or rVV-β-gal *via* scarification, and 14 days later they were challenged with 3 × 10^6^ PFU human RSV intranasally	Mice previously infected (Flu-G-RSV or RSV-G-RSV) exhibit decreased eosinophilia and weight loss (compared with Hep-2-G-RSV)	Flu-G-RSV mice had decreased TNF-α and IL-4 cytokine levels. In addition, 16.9 ± 2.7% of CD8 T cells recruited into the lung (post RSV infection) bound influenza tetramer, and 39.4 ± 3.8% expressed IFN-γ. Transfer of splenocytes at 21 or 149 days post influenza virus infection, followed by rVV-G and RSV challenge 14 days later also resulted in decreased eosinophilia	(35)

Acute, sequential	C57BL/6, B6.Pl-*Thy1^a^*/Cy (Thy1.1) and B6.SJL*ptprc^a^pep3^b^*/BoyJ (CD45.1)	Sendai virus (enders strain)	Influenza virus (X31 and H3N2)	Mice were infected with 250 EID_50_ Sendai virus and challenged 30–35 days later with 300 EID_50_ X31. For reverse order, mice were infected with 300 EID_50_ of X31 and challenged with 250 EID_50_ of Sendai virus 30–35 days post flu infection	Requires further investigation	Early infiltration and ~5× increase in cell number of Sendai virus specific CD8 T cells into the lungs of flu infected mice (day 4 post flu). Flu specific [nucleoprotein (NP) and polymerase acidic protein (PA)] CD8 T cell responses were unaltered, and early recruitment of memory cells was from migration of cells from other anatomical sites. When the sequence of infection was reversed, early infiltration and ~4× increase of flu specific memory CD8s occurred at day 4 post Sendai virus infection	(46)

Acute, sequential	Human	Influenza virus	Acute Epstein–Barr virus (EBV)	Influenza A virus-immune patients with acute EBV infection were recruited from the Umass Student Health Services. Age ranged from 18 to 23 years old. Acute EBV infection was confirmed using a monospot test and detection of anti-EBV capsid IgM in patient sera. Healthy volunteers were recruited from UMass Medical School. Age ranged from 24 to 50 years old	See Ref. (61)	Identified cross-reactive CD8 T cells specific for influenza A virus M1_58_ and EBV-BMLF1_280_, despite only 33% sequence homology	(59)

Acute, sequential	Human	Influenza virus	Acute EBV	Influenza A virus-immune patients with acute EBV infection were recruited from the Umass Student Health Services. Age ranged from 18 to 23 years old. Acute EBV infection was confirmed using a monospot test and detection of anti-EBV capsid IgM in patient sera. Healthy volunteers were recruited from UMass Medical School. Age ranged from 42 to 50 years old	See Ref. (61)	Cross-reactive M1 and BMLF-specific CD8 T cells utilize unique clones not found in single M1 or BMLF-specific CD8 T cell pools. Computer simulation suggests the effects of cross-reactivity on T cell receptor (TCR) repertoire diversity depends on the degree of similarity between epitopes. If epitopes are structurally similar, termed “near cross-reactive,” responses will lead to a more narrow TCR repertoire, whereas cross-reactive responses between structurally divergent epitopes, termed “far cross-reactive,” will lead to a broad TCR repertoire	(60)

Acute, sequential	Human	Influenza virus	Acute EBV	College students with symptoms of acute infectious mononucleosis (AIM) were recruited. Age ranged from 18 to 30 years old. Acute EBV infection was confirmed by a monospot test and the detection of anti-EBV capsid IgM in patient serum. Healthy, EBV-seropositive donors, age >18 years old, were used as controls	CD8 T cells cross-reactive for influenza and EBV epitopes may contribute to AIM disease severity by augmenting CD8 T cell responses.	IAV-M1+/EBV-BMLF+ double positive CD8 T cells had the strongest correlation with AIM disease severity and predict severe AIM in a relative-risk analysis. Single IAV-M1 and EBV-BMLF each had weaker associations and no other tetramer + population tested (2 two from CMV and EBV) were correlated with AIM severity	(61)

Acute, sequential and chronic co-infection	C57BL/6	Influenza virus	Acute and chronic murine herpesvirus 68 (MHV68)	Mice were primed with 10^7.9^ EID_50_ PR8 intraperitoneally, challenged with 10^6.5^ EID_50_ X31 intranasally, and later were or were not infected with 10^4^ PFU of MHV68 intranasally. In another study, mice were infected intranasally with MHV68, boosted intraperitoneally with 5 × 10^7^ PFU of recombinant VV expressing MHV86 p56 peptide AGPHNDMEI (Vacc-p56), and then were or were not challenged with X31 intranasally. Each infection was delivered 6 weeks apart	Requires further investigation	Co-infected mice (PR8-X31-MHV68) show attrition of influenza (NP) and MHV68 (p79)-specific memory CD8 T cells compared with their respective single infected counterparts at day 100. The presence and degree of attrition varies by anatomical site in both cases. In addition, mice primed with MHV68 then sequentially infected with influenza virus (MHV68-vacc-p56-X31) exhibit higher numbers of influenza-specific CD8 T cells at day 14, but a lower number at day 200	(79)

Chronic co-infection	BALB/c	MHV68 (WUMS strain)	Influenza virus (PR8 and H1N1)	Mice were infected with 4 × 10^4^ PFU MHV68 or PBS (mock-infected) and 28, 60, or 120 days later were challenged with 1 × 10^4^ PFU PR8	Latent MHV68 infection confers protection against influenza virus challenge, as determined by improved survival, enhanced influenza viral clearance, and decreased lung injury	Co-infected mice exhibit increased levels of IFN-γ, TNF-α, and IL-12p40, but decreased levels of neutrophil chemokines CXCL1 (KC) and CXCL2 (MIP-2α). Co-infected mice also had increased numbers of CD69+ CD4+ (day 0 and 4) and CD8+ T (day 0, 4, and 6) cells in the lung, decreased neutrophils (day 8), and enhanced activation of alveolar macrophages. Adoptive transfer of macrophages from co-infected mice was sufficient to confer protection against influenza virus challenge	(47)

Chronic co-infection	C57BL/6 and IFN-γ KO	Cytomegalovirus (Smith)	Influenza virus (X31 and H3N2)	Mice were infected with 4 × 10^4^ PFU of murine cytomegalovirus (MCMV) intraperitoneally. Co-infected mice were also infected with 1 × 10^6^ EID_50_ of X31 at 5 weeks (early latency), 12 weeks (established latency), or 9 months (long-standing latency) post MCMV infection	MCMV co-infection confers protection against influenza virus challenge, but protection wanes with time and is not observed in long-standing latent MCMV infection	Mice co-infected with influenza virus at 5 or 12 weeks post MCMV infection exhibit higher influenza-specific CD8 T cell responses against three immunodominant influenza epitopes (polymerase basic protein 1, PA, and NP) and decreased influenza virus titers	(21)

Chronic co-infection	Human	Cytomegalovirus	Fluzone vaccine (each dose contains 15 µg of HA from H1N1, H3N2, and B strains)	Ninety-one healthy donors were enrolled at the Stanford-LPCH Vaccine Program in fall of 2008 (89 completed the study). The validation cohort consisted of 77 individuals who returned in fall of 2009, plus 37 subjects vaccinated in another study between 2010 and 2011 flu seasons	Young CMV seropositive subjects had higher antibody response to the Fluzone vaccine at 28 days and 1 year post vaccination, as compared with young, CMV seronegative subjects. However, no difference was observed in the elderly, based on CMV serostatus	Young CMV+ subjects have a broadly activated immune system compared with their CMV-counterparts. This is reflected by augmented expression of genes important for immune activation (e.g., antigen processing and presentation, NK cell cytotoxicity), increased levels of IL-13, IFN-γ, and CD8+ pSTAT1/3 in response to IL-6 stimulation. This study also found, elderly CMV+ subjects showed lower responses to IL-6, compared with young CMV+	(21)

Chronic co-infection	Human	Human cytomegalovirus (HCMV) and/or EBV	Influenza virus	Samples were collected from 50 patients [20 hepatitis B virus (HBV), 12 influenza, 12 dengue, 3 adenovirus, 3 fevers with unknown etiology] and 5 healthy volunteers attending clinics in Singapore or Italy. Diagnosis was confirmed utilizing appropriate methods for the infection within 5 days of selection. For example, influenza infections were confirmed with isolation of influenza A virus from nasal swabs	Requires further investigation	Acute infection with influenza, HBV, dengue, and adenovirus induce activation (CD38+ HLA-DR+) of HCMV- and EBV-specific CD8 T cells. In one influenza patient, 1/4 of activated CD8 T cells at onset were HCMV-specific, and influenza-specific CD8 T cells could not be detected until day 5. In addition, IL-15 preferentially activates memory CD8 T cells specific for chronic infections, augments anti-viral cytokine production with TCR stimulation, and is sufficient for spontaneous IFN-γ production	(50)

## Author Contributions

PT assisted in planning and editing the review. AS planned and wrote the review, and designed and created the figure.

## Conflict of Interest Statement

The authors declare that the research was conducted in the absence of any commercial or financial relationships that could be construed as a potential conflict of interest.

## References

[1] Molinari N-AM Ortega-Sanchez IR Messonnier ML Thompson WW Wortley PM Weintraub E The annual impact of seasonal influenza in the US: measuring disease burden and costs. Vaccine (2007) 25:5086–96. 10.1016/j.vaccine.2007.03.046 17544181

[2] Taubenberger JK Kash JC. Influenza virus evolution, host adaptation, and pandemic formation. Cell Host Microbe (2010) 7:440–51. 10.1016/j.chom.2010.05.009 20542248 PMC2892379

[3] Duan S Thomas PG. Balancing immune protection and immune pathology by CD8(+) T-cell responses to influenza infection. Front Immunol (2016) 7:25. 10.3389/fimmu.2016.00025 26904022 PMC4742794

[4] Yewdell JW Bennink JR. Immunodominance in major histocompatibility complex class I-restricted T lymphocyte responses. Annu Rev Immunol (1999) 17:51–88. 10.1146/annurev.immunol.17.1.51 10358753

[5] Cukalac T Chadderton J Zeng W Cullen JG Kan WT Doherty PC The influenza virus-specific CTL immunodominance hierarchy in mice is determined by the relative frequency of high-avidity T cells. J Immunol (2014) 192:4061–8. 10.4049/jimmunol.1301403 24696232

[6] La Gruta NL Rothwell WT Cukalac T Swan NG Valkenburg SA Kedzierska K Primary CTL response magnitude in mice is determined by the extent of naive T cell recruitment and subsequent clonal expansion. J Clin Invest (2010) 120:1885–94. 10.1172/JCI41538 20440073 PMC2877949

[7] Crowe SR Turner SJ Miller SC Roberts AD Rappolo RA Doherty PC Differential antigen presentation regulates the changing patterns of CD8+ T cell immunodominance in primary and secondary influenza virus infections. J Exp Med (2003) 198:399–410. 10.1084/jem.20022151 12885871 PMC2194086

[8] Davis MM Bjorkman PJ. T-cell antigen receptor genes and T-cell recognition. Nature (1988) 334:395. 10.1038/334395a0 3043226

[9] Marcou Q Mora T Walczak AM. High-throughput immune repertoire analysis with IGoR. Nat Commun (2018) 9:561. 10.1038/s41467-018-02832-w 29422654 PMC5805751

[10] Mora T Walczak A Quantifying lymphocyte receptor diversity. bioRxiv (2016) 046870 10.1101/046870

[11] Arstila TP Casrouge A Baron V Even J Kanellopoulos J Kourilsky P. A direct estimate of the human alphabeta T cell receptor diversity. Science (1999) 286:958–61. 10.1126/science.286.5441.958 10542151

[12] Casrouge A Beaudoing E Dalle S Pannetier C Kanellopoulos J Kourilsky P. Size estimate of the alpha beta TCR repertoire of naive mouse splenocytes. J Immunol (2000) 164:5782–7. 10.4049/jimmunol.164.11.5782 10820256

[13] Robins HS Campregher PV Srivastava SK Wacher A Turtle CJ Kahsai O Comprehensive assessment of T-cell receptor β-chain diversity in αβ T cells. Blood (2009) 114:4099–107. 10.1182/blood-2009-04-217604 19706884 PMC2774550

[14] Warren RL Freeman JD Zeng T Choe G Munro S Moore R Exhaustive T-cell repertoire sequencing of human peripheral blood samples reveals signatures of antigen selection and a directly measured repertoire size of at least 1 million clonotypes. Genome Res (2011) 21:790–7. 10.1101/gr.115428.110 21349924 PMC3083096

[15] Moon JJ Chu HH Pepper M McSorley SJ Jameson SC Kedl RM Naive CD4(+) T cell frequency varies for different epitopes and predicts repertoire diversity and response magnitude. Immunity (2007) 27:203–13. 10.1016/j.immuni.2007.07.007 17707129 PMC2200089

[16] Obar JJ Khanna KM Lefrançois L. Endogenous naive CD8+ T cell precursor frequency regulates primary and memory responses to infection. Immunity (2008) 28:859–69. 10.1016/j.immuni.2008.04.010 18499487 PMC2836785

[17] Wherry EJ Ahmed R Memory CD8 T-cell differentiation during viral infection. J Virol (2004) 78:5535–45. 10.1128/JVI.78.11.5535-5545.2004 15140950 PMC415833

[18] Youngblood B Hale JS Ahmed R. T-cell memory differentiation: insights from transcriptional signatures and epigenetics. Immunology (2013) 139:277–84. 10.1111/imm.12074 23347146 PMC3701173

[19] Virgin HW Wherry EJ Ahmed R. Redefining chronic viral infection. Cell (2009) 138:30–50. 10.1016/j.cell.2009.06.036 19596234

[20] Reese TA Bi K Kambal A Filali-Mouhim A Beura LK Bürger MC Sequential infection with common pathogens promotes human-like immune gene expression and altered vaccine response. Cell Host Microbe (2016) 19:713–9. 10.1016/j.chom.2016.04.003 27107939 PMC4896745

[21] Furman D Jojic V Sharma S Shen-Orr SS Angel CJL Onengut-Gumuscu S Cytomegalovirus infection enhances the immune response to influenza. Sci Transl Med (2015) 7:281ra43. 10.1126/scitranslmed.aaa2293 25834109 PMC4505610

[22] Grant EJ Quiñones-Parra SM Clemens EB Kedzierska K. Human influenza viruses and CD8(+) T cell responses. Curr Opin Virol (2016) 16:132–42. 10.1016/j.coviro.2016.01.016 26974887

[23] Van Epps HL Terajima M Mustonen J Arstila TP Corey EA Vaheri A Long-lived memory T lymphocyte responses after hantavirus infection. J Exp Med (2002) 196:579–88. 10.1084/jem.20011255 12208874 PMC2194003

[24] Wang Z Loh L Kedzierski L Kedzierska K. Avian influenza viruses, inflammation, and CD8(+) T cell immunity. Front Immunol (2016) 7:60. 10.3389/fimmu.2016.00060 26973644 PMC4771736

[25] Callan MFC. The evolution of antigen-specific CD8+ T cell responses after natural primary infection of humans with Epstein-Barr virus. Viral Immunol (2003) 16:3–16. 10.1089/088282403763635401 12725684

[26] Laichalk LL Thorley-Lawson DA. Terminal differentiation into plasma cells initiates the replicative cycle of Epstein-Barr virus in vivo. J Virol (2005) 79:1296–307. 10.1128/JVI.79.2.1296-1307.2005 15613356 PMC538585

[27] Torti N Oxenius A. T cell memory in the context of persistent herpes viral infections. Viruses (2012) 4:1116–43. 10.3390/v4071116 22852044 PMC3407898

[28] Sandberg JK Fast NM Nixon DF. Functional heterogeneity of cytokines and cytolytic effector molecules in human CD8+ T lymphocytes. J Immunol (2001) 167:181–7. 10.4049/jimmunol.167.1.181 11418647

[29] Souquette A Frere J Smithey M Sauce D Thomas PG. A constant companion: immune recognition and response to cytomegalovirus with aging and implications for immune fitness. Geroscience (2017) 39:293–303. 10.1007/s11357-017-9982-x 28647907 PMC5505896

[30] Claassen MAA Janssen HLA Boonstra A. Role of T cell immunity in hepatitis C virus infections. Curr Opin Virol (2013) 3:461–7. 10.1016/j.coviro.2013.05.006 23735335

[31] Douek DC Picker LJ Koup RA. T cell dynamics in HIV-1 infection. Annu Rev Immunol (2003) 21:265–304. 10.1146/annurev.immunol.21.120601.141053 12524385

[32] Holz L Rehermann B. T cell responses in hepatitis C virus infection: historical overview and goals for future research. Antiviral Res (2015) 114:96–105. 10.1016/j.antiviral.2014.11.009 25433310 PMC4480227

[33] Streeck H Nixon DF. T cell immunity in acute HIV-1 infection. J Infect Dis (2010) 202(Suppl 2):S302–8. 10.1086/655652 20846037 PMC2954287

[34] Walker B McMichael A The T-cell response to HIV. Cold Spring Harb Perspect Med (2012) 2:a007054 10.1101/cshperspect.a007054 23002014 PMC3543107

[35] Walzl G Tafuro S Moss P Openshaw PJ Hussell T. Influenza virus lung infection protects from respiratory syncytial virus-induced immunopathology. J Exp Med (2000) 192:1317–26. 10.1084/jem.192.9.1317 11067880 PMC2193356

[36] Welsh RM Selin LK. No one is naive: the significance of heterologous T-cell immunity. Nat Rev Immunol (2002) 2:417–26. 10.1038/nri820 12093008

[37] Ehl S Hombach J Aichele P Hengartner H Zinkernagel RM. Bystander activation of cytotoxic T cells: studies on the mechanism and evaluation of in vivo significance in a transgenic mouse model. J Exp Med (1997) 185:1241–51. 10.1084/jem.185.7.1241 9104811 PMC2196250

[38] Tough DF Borrow P Sprent J. Induction of bystander T cell proliferation by viruses and type I interferon in vivo. Science (1996) 272:1947–50. 10.1126/science.272.5270.1947 8658169

[39] Freeman BE Hammarlund E Raué H-P Slifka MK. Regulation of innate CD8+ T-cell activation mediated by cytokines. Proc Natl Acad Sci U S A (2012) 109:9971–6. 10.1073/pnas.1203543109 22665806 PMC3382521

[40] Gilbertson B Germano S Steele P Turner S Fazekas de St Groth B Cheers C. Bystander activation of CD8+ T lymphocytes during experimental mycobacterial infection. Infect Immun (2004) 72:6884–91. 10.1128/IAI.72.12.6884-6891.2004 15557609 PMC529149

[41] Martin MD Badovinac VP. Antigen-dependent and -independent contributions to primary memory CD8 T cell activation and protection following infection. Sci Rep (2015) 5:18022. 10.1038/srep18022 26658291 PMC4675085

[42] Sckisel GD Tietze JK Zamora AE Hsiao H-H Priest SO Wilkins DEC Influenza infection results in local expansion of memory CD8(+) T cells with antigen non-specific phenotype and function. Clin Exp Immunol (2014) 175:79–91. 10.1111/cei.12186 23937663 PMC3898557

[43] Chu T Tyznik AJ Roepke S Berkley AM Woodward-Davis A Pattacini L Bystander-activated memory CD8 T cells control early pathogen load in an innate-like, NKG2D-dependent manner. Cell Rep (2013) 3:701–8. 10.1016/j.celrep.2013.02.020 23523350 PMC3628815

[44] Verneris MR Karami M Baker J Jayaswal A Negrin RS. Role of NKG2D signaling in the cytotoxicity of activated and expanded CD8 T cells. Blood (2004) 103(8):3065–72. 10.1182/blood-2003-06-2125 15070686

[45] Zloza A Kohlhapp FJ Lyons GE Schenkel JM Moore TV Lacek AT NKG2D signaling on CD8+ T cells represses T-bet and rescues CD4-unhelped CD8+ T cell memory recall but not effector responses. Nat Med (2012) 18:422–8. 10.1038/nm.2683 22366950 PMC3436127

[46] Ely KH Cauley LS Roberts AD Brennan JW Cookenham T Woodland DL. Nonspecific recruitment of memory CD8+ T cells to the lung airways during respiratory virus infections. J Immunol (2003) 170:1423–9. 10.4049/jimmunol.170.3.1423 12538703

[47] Saito F Ito T Connett JM Schaller MA Carson WF IV Hogaboam CM MHV68 latency modulates the host immune response to influenza A virus. Inflammation (2013) 36:1295–303. 10.1007/s10753-013-9668-1 23807051 PMC3825492

[48] Bate SL Dollard SC Cannon MJ Cytomegalovirus seroprevalence in the United States: the national health and nutrition examination surveys, 1988–2004. Clin Infect Dis (2010) 50(11):1439–47. 10.1086/652438 20426575 PMC11000537

[49] Pawelec G McElhaney JE Aiello AE Derhovanessian E. The impact of CMV infection on survival in older humans. Curr Opin Immunol (2012) 24:507–11. 10.1016/j.coi.2012.04.002 22541724

[50] Sandalova E Laccabue D Boni C Tan AT Fink K Ooi EE Contribution of herpesvirus specific CD8 T cells to anti-viral T cell response in humans. PLoS Pathog (2010) 6:e1001051. 10.1371/journal.ppat.1001051 20808900 PMC2924358

[51] Mason D A very high level of crossreactivity is an essential feature of the T-cell receptor. Immunol Today (1998) 19:395–404. 10.1016/S0167-5699(98)01299-7 9745202

[52] Duan S Meliopoulos VA McClaren JL Guo X-ZJ Sanders CJ Smallwood HS Diverse heterologous primary infections radically alter immunodominance hierarchies and clinical outcomes following H7N9 influenza challenge in mice. PLoS Pathog (2015) 11:e1004642. 10.1371/journal.ppat.1004642 25668410 PMC4335497

[53] Kim S-K Brehm MA Welsh RM Selin LK. Dynamics of memory T cell proliferation under conditions of heterologous immunity and bystander stimulation. J Immunol (2002) 169:90–8. 10.4049/jimmunol.169.1.90 12077233

[54] Brehm MA Pinto AK Daniels KA Schneck JP Welsh RM Selin LK. T cell immunodominance and maintenance of memory regulated by unexpectedly cross-reactive pathogens. Nat Immunol (2002) 3:627–34. 10.1038/ni806 12055626

[55] Alanio C Nicoli F Sultanik P Flecken T Perot B Duffy D Bystander hyperactivation of preimmune CD8+ T cells in chronic HCV patients. Elife (2015) 4:e07916. 10.7554/eLife.07916 26568315 PMC4752008

[56] Che JW Daniels KA Selin LK Welsh RM. Heterologous immunity and persistent murine cytomegalovirus infection. J Virol (2017) 91:e01386-16. 10.1128/JVI.01386-16 27807227 PMC5215326

[57] Stelekati E Shin H Doering TA Dolfi DV Ziegler CG Beiting DP Bystander chronic infection negatively impacts development of CD8(+) T cell memory. Immunity (2014) 40:801–13. 10.1016/j.immuni.2014.04.010 24837104 PMC4114317

[58] Wedemeyer H Mizukoshi E Davis AR Bennink JR Rehermann B. Cross-reactivity between hepatitis C virus and Influenza A virus determinant-specific cytotoxic T cells. J Virol (2001) 75:11392–400. 10.1128/JVI.75.23.11392-11400.2001 11689620 PMC114725

[59] Clute SC Watkin LB Cornberg M Naumov YN Sullivan JL Luzuriaga K Cross-reactive influenza virus-specific CD8+ T cells contribute to lymphoproliferation in Epstein-Barr virus-associated infectious mononucleosis. J Clin Invest (2005) 115:3602–12. 10.1172/JCI25078 16308574 PMC1288832

[60] Clute SC Naumov YN Watkin LB Aslan N Sullivan JL Thorley-Lawson DA Broad cross-reactive TCR repertoires recognizing dissimilar Epstein-Barr and influenza A virus epitopes. J Immunol (2010) 185:6753–64. 10.4049/jimmunol.1000812 21048112 PMC3738202

[61] Aslan N Watkin LB Gil A Mishra R Clark FG Welsh RM Severity of acute infectious mononucleosis correlates with cross-reactive influenza CD8 T-cell receptor repertoires. MBio (2017) 8:e1841–1817. 10.1128/mBio.01841-17 29208744 PMC5717389

[62] Ely KH Roberts AD Kohlmeier JE Blackman MA Woodland DL. Aging and CD8+ T cell immunity to respiratory virus infections. Exp Gerontol (2007) 42:427–31. 10.1016/j.exger.2006.11.017 17197143 PMC1964788

[63] Thompson WW Weintraub E Dhankhar P Cheng P-Y Brammer L Meltzer MI Estimates of US influenza-associated deaths made using four different methods. Influenza Other Respir Viruses (2009) 3:37–49. 10.1111/j.1750-2659.2009.00073.x 19453440 PMC4986622

[64] Blackman MA Woodland DL. The narrowing of the CD8 T cell repertoire in old age. Curr Opin Immunol (2011) 23:537–42. 10.1016/j.coi.2011.05.005 21652194 PMC3163762

[65] Moreau J-F Pradeu T Grignolio A Nardini C Castiglione F Tieri P The emerging role of ECM crosslinking in T cell mobility as a hallmark of immunosenescence in humans. Ageing Res Rev (2017) 35:322–35. 10.1016/j.arr.2016.11.005 27876574

[66] Nikolich-Žugich J Li G Uhrlaub JL Renkema KR Smithey MJ. Age-related changes in CD8 T cell homeostasis and immunity to infection. Semin Immunol (2012) 24:356–64. 10.1016/j.smim.2012.04.009 22554418 PMC3480557

[67] Shaw AC Goldstein DR Montgomery RR. Age-dependent dysregulation of innate immunity. Nat Rev Immunol (2013) 13:875–87. 10.1038/nri3547 24157572 PMC4096436

[68] Khan N Shariff N Cobbold M Bruton R Ainsworth JA Sinclair AJ Cytomegalovirus seropositivity drives the CD8 T cell repertoire toward greater clonality in healthy elderly individuals. J Immunol (2002) 169:1984–92. 10.4049/jimmunol.169.4.1984 12165524

[69] Klenerman P Hill A. T cells and viral persistence: lessons from diverse infections. Nat Immunol (2005) 6:873–9. 10.1038/ni1241 16116467

[70] Whiting CC Siebert J Newman AM Du H-W Alizadeh AA Goronzy J Large-scale and comprehensive immune profiling and functional analysis of normal human aging. PLoS One (2015) 10:e0133627. 10.1371/journal.pone.0133627 26197454 PMC4509650

[71] Vezys V Yates A Casey KA Lanier G Ahmed R Antia R Memory CD8 T-cell compartment grows in size with immunological experience. Nature (2009) 457:196–9. 10.1038/nature07486 19005468

[72] Selin LK Welsh RM. Plasticity of T cell memory responses to viruses. Immunity (2004) 20:5–16. 10.1016/S1074-7613(03)00356-X 14738760 PMC7130098

[73] Bahl K Kim S-K Calcagno C Ghersi D Puzone R Celada F IFN-induced attrition of CD8 T cells in the presence or absence of cognate antigen during the early stages of viral infections. J Immunol (2006) 176:4284–95. 10.4049/jimmunol.176.7.4284 16547266

[74] McNally JM Zarozinski CC Lin MY Brehm MA Chen HD Welsh RM. Attrition of bystander CD8 T cells during virus-induced T-cell and interferon responses. J Virol (2001) 75:5965–76. 10.1128/JVI.75.13.5965-5976.2001 11390598 PMC114312

[75] Jiang J Gross D Nogusa S Elbaum P Murasko DM. Depletion of T cells by type I interferon: differences between young and aged mice. J Immunol (2005) 175:1820–6. 10.4049/jimmunol.175.3.1820 16034124

[76] Selin LK Vergilis K Welsh RM Nahill SR. Reduction of otherwise remarkably stable virus-specific cytotoxic T lymphocyte memory by heterologous viral infections. J Exp Med (1996) 183:2489–99. 10.1084/jem.183.6.2489 8676069 PMC2192604

[77] Jiang J Lau LL Shen H. Selective depletion of nonspecific T cells during the early stage of immune responses to infection. J Immunol (2003) 171:4352–8. 10.4049/jimmunol.171.8.4352 14530360

[78] Selin LK Lin MY Kraemer KA Pardoll DM Schneck JP Varga SM Attrition of T cell memory: selective loss of LCMV epitope-specific memory CD8 T cells following infections with heterologous viruses. Immunity (1999) 11:733–42. 10.1016/S1074-7613(00)80147-8 10626895

[79] Liu H Andreansky S Diaz G Turner SJ Wodarz D Doherty PC. Quantitative analysis of long-term virus-specific CD8+-T-cell memory in mice challenged with unrelated pathogens. J Virol (2003) 77:7756–63. 10.1128/JVI.77.14.7756-7763.2003 12829815 PMC161950

[80] Kim S-K Welsh RM. Comprehensive early and lasting loss of memory CD8 T cells and functional memory during acute and persistent viral infections. The Journal of Immunology (2004) 172:3139–50. 10.4049/jimmunol.172.5.3139 14978120

[81] Sckisel GD Bouchlaka MN Monjazeb AM Crittenden M Curti BD Wilkins DEC Out-of-sequence signal 3 paralyzes primary CD4(+) T-cell-dependent immunity. Immunity (2015) 43:240–50. 10.1016/j.immuni.2015.06.023 26231116 PMC4770886

[82] Danahy DB Anthony SM Jensen IJ Hartwig SM Shan Q Xue H-H Polymicrobial sepsis impairs bystander recruitment of effector cells to infected skin despite optimal sensing and alarming function of skin resident memory CD8 T cells. PLoS Pathog (2017) 13:e1006569. 10.1371/journal.ppat.1006569 28910403 PMC5599054

[83] Beverley PC. Is T-cell memory maintained by crossreactive stimulation? Immunol Today (1990) 11:203–5. 10.1016/0167-5699(90)90083-L 2191683

[84] Nahill SR Welsh RM. High frequency of cross-reactive cytotoxic T lymphocytes elicited during the virus-induced polyclonal cytotoxic T lymphocyte response. J Exp Med (1993) 177:317–27. 10.1084/jem.177.2.317 8093891 PMC2190893

[85] Smith DK Dudani R Pedras-Vasconcelos JA Chapdelaine Y van Faassen H Sad S. Cross-reactive antigen is required to prevent erosion of established T cell memory and tumor immunity: a heterologous bacterial model of attrition. J Immunol (2002) 169:1197–206. 10.4049/jimmunol.169.3.1197 12133940

[86] Purwar R Campbell J Murphy G Richards WG Clark RA Kupper TS. Resident memory T cells (T(RM)) are abundant in human lung: diversity, function, and antigen specificity. PLoS One (2011) 6:e16245. 10.1371/journal.pone.0016245 21298112 PMC3027667

[87] Pizzolla A Nguyen TH Sant S Jaffar J Loudovaris T Mannering SI Influenza-specific lung-resident memory T cells are proliferative and polyfunctional and maintain diverse TCR profiles. J Clin Invest (2018) 128:721–33. 10.1172/JCI96957 29309047 PMC5785253

[88] Hombrink P Helbig C Backer RA Piet B Oja AE Stark R Programs for the persistence, vigilance and control of human CD8+ lung-resident memory T cells. Nat Immunol (2016) 17:1467. 10.1038/ni.3589 27776108

[89] Kohlmeier JE Cookenham T Roberts AD Miller SC Woodland DL. Type I interferons regulate cytolytic activity of memory CD8(+) T cells in the lung airways during respiratory virus challenge. Immunity (2010) 33:96–105. 10.1016/j.immuni.2010.06.016 20637658 PMC2908370

[90] López-Cabrera M Muñoz E Blázquez MV Transcriptional regulation of the gene encoding the human C-type lectin leukocyte receptor AIM/CD69 and functional characterization of its tumor necrosis factor-α-responsive elements. J Biol Chem (1995) 270(37):21545–51. 10.1074/jbc.270.37.21545 7665567

[91] Schenkel JM Masopust D. Tissue-resident memory T cells. Immunity (2014) 41:886–97. 10.1016/j.immuni.2014.12.007 25526304 PMC4276131

[92] Mackay LK Rahimpour A Ma JZ Collins N Stock AT Hafon M-L The developmental pathway for CD103+CD8+ tissue-resident memory T cells of skin. Nat Immunol (2013) 14:1294 10.1038/ni.2744 24162776

[93] Mueller SN Mackay LK. Tissue-resident memory T cells: local specialists in immune defence. Nat Rev Immunol (2016) 16:79–89. 10.1038/nri.2015.3 26688350

[94] Piet B de Bree GJ Smids-Dierdorp BS van der Loos CM Remmerswaal EBM von der Thüsen JH CD8+ T cells with an intraepithelial phenotype upregulate cytotoxic function upon influenza infection in human lung. J Clin Invest (2011) 121:2254–63. 10.1172/JCI44675 21537083 PMC3104744

[95] Turner DL Bickham KL Thome JJ Kim CY D’Ovidio F Wherry EJ Lung niches for the generation and maintenance of tissue-resident memory T cells. Mucosal Immunol (2014) 7:501–10. 10.1038/mi.2013.67 24064670 PMC3965651

[96] de Bree GJ van Leeuwen EMM Out TA Jansen HM Jonkers RE van Lier RAW. Selective accumulation of differentiated CD8+ T cells specific for respiratory viruses in the human lung. J Exp Med (2005) 202:1433–42. 10.1084/jem.20051365 16301748 PMC2212987

[97] Ariotti S Hogenbirk MA Dijkgraaf FE Visser LL Hoekstra ME Song J-Y T cell memory. Skin-resident memory CD8+ T cells trigger a state of tissue-wide pathogen alert. Science (2014) 346:101–5. 10.1126/science.1254803 25278612

[98] Jiang X Clark RA Liu L Wagers AJ Fuhlbrigge RC Kupper TS. Skin infection generates non-migratory memory CD8+ T(RM) cells providing global skin immunity. Nature (2012) 483:227–31. 10.1038/nature10851 22388819 PMC3437663

[99] Schenkel JM Fraser KA Beura LK Pauken KE Vezys V Masopust D. T cell memory. Resident memory CD8 T cells trigger protective innate and adaptive immune responses. Science (2014) 346:98–101. 10.1126/science.1254536 25170049 PMC4449618

[100] Teijaro JR Turner D Pham Q Wherry EJ Lefrançois L Farber DL. Cutting edge: tissue-retentive lung memory CD4 T cells mediate optimal protection to respiratory virus infection. J Immunol (2011) 187:5510–4. 10.4049/jimmunol.1102243 22058417 PMC3221837

[101] Wakim LM Gupta N Mintern JD Villadangos JA Enhanced survival of lung tissue-resident memory CD8+ T cells during infection with influenza virus due to selective expression of IFITM3. Nat Immunol (2013) 14:238 10.1038/ni.2525 23354485

[102] Lee Y-N Lee Y-T Kim M-C Gewirtz AT Kang S-M. A novel vaccination strategy mediating the induction of lung-resident memory CD8 T cells confers heterosubtypic immunity against future pandemic influenza virus. J Immunol (2016) 196:2637–45. 10.4049/jimmunol.1501637 26864033 PMC4779729

[103] McMaster SR Wilson JJ Wang H Kohlmeier JE Airway-resident memory CD8 T cells provide antigen-specific protection against respiratory virus challenge through rapid IFN-γ production. J Immunol (2015) 195:203–9. 10.4049/jimmunol.1402975 26026054 PMC4475417

[104] Pizzolla A Nguyen THO Smith JM Brooks AG Kedzieska K Heath WR Resident memory CD8+ T cells in the upper respiratory tract prevent pulmonary influenza virus infection. Sci Immunol (2017) 2:eaam6970. 10.1126/sciimmunol.aam6970 28783656

[105] Wakim LM Smith J Caminschi I Lahoud MH Villadangos JA. Antibody-targeted vaccination to lung dendritic cells generates tissue-resident memory CD8 T cells that are highly protective against influenza virus infection. Mucosal Immunol (2015) 8:1060–71. 10.1038/mi.2014.133 25586557

[106] Wu T Hu Y Lee Y-T Bouchard KR Benechet A Khanna K Lung-resident memory CD8 T cells (TRM) are indispensable for optimal cross-protection against pulmonary virus infection. J Leukoc Biol (2014) 95:215–24. 10.1189/jlb.0313180 24006506 PMC3896663

[107] Zens KD Chen JK Farber DL. Vaccine-generated lung tissue-resident memory T cells provide heterosubtypic protection to influenza infection. JCI Insight (2016) 1:e85832. 10.1172/jci.insight.85832 27468427 PMC4959801

[108] Zhou AC Wagar LE Wortzman ME Watts TH. Intrinsic 4-1BB signals are indispensable for the establishment of an influenza-specific tissue-resident memory CD8 T-cell population in the lung. Mucosal Immunol (2017) 10:1294–309. 10.1038/mi.2016.124 28051085

[109] Laidlaw BJ Zhang N Marshall HD Staron MM Guan T Hu Y CD4+ T cell help guides formation of CD103+ lung-resident memory CD8+ T cells during influenza viral infection. Immunity (2014) 41:633–45. 10.1016/j.immuni.2014.09.007 25308332 PMC4324721

[110] Smith CM Wilson NS Waithman J Villadangos JA Carbone FR Heath WR Cognate CD4(+) T cell licensing of dendritic cells in CD8(+) T cell immunity. Nat Immunol (2004) 5:1143–8. 10.1038/ni1129 15475958

[111] Yu CI Becker C Wang Y Marches F Helft J Leboeuf M Human CD1c+ dendritic cells drive the differentiation of CD103+ CD8+ mucosal effector T cells via the cytokine TGF-β. Immunity (2013) 38:818–30. 10.1016/j.immuni.2013.03.004 23562160 PMC3639491

[112] Kim TS Braciale TJ. Respiratory dendritic cell subsets differ in their capacity to support the induction of virus-specific cytotoxic CD8+ T cell responses. PLoS One (2009) 4:e4204. 10.1371/journal.pone.0004204 19145246 PMC2615220

[113] Kim TS Gorski SA Hahn S Murphy KM Braciale TJ. Distinct dendritic cell subsets dictate the fate decision between effector and memory CD8(+) T cell differentiation by a CD24-dependent mechanism. Immunity (2014) 40:400–13. 10.1016/j.immuni.2014.02.004 24631155 PMC4017923

[114] Yoshizawa A Bi K Keskin DB Zhang G Reinhold B Reinherz EL TCR-pMHC encounter differentially regulates transcriptomes of tissue-resident CD8 T cells. Eur J Immunol (2018) 48:128–50. 10.1002/eji.201747174 28872670 PMC6531858

[115] Slütter B Van Braeckel-Budimir N Abboud G Varga SM Salek-Ardakani S Harty JT. Dynamics of influenza-induced lung-resident memory T cells underlie waning heterosubtypic immunity. Sci Immunol (2017) 2:eaag2031. 10.1126/sciimmunol.aag2031 28783666 PMC5590757

[116] Barton ES White DW Cathelyn JS Brett-McClellan KA Engle M Diamond MS Herpesvirus latency confers symbiotic protection from bacterial infection. Nature (2007) 447:326–9. 10.1038/nature05762 17507983

[117] Cornberg M Chen AT Wilkinson LA Brehm MA Kim S-K Calcagno C Narrowed TCR repertoire and viral escape as a consequence of heterologous immunity. J Clin Invest (2006) 116:1443–56. 10.1172/JCI27804 16614754 PMC1435724

[118] Schmidt ME Knudson CJ Hartwig SM Pewe LL Meyerholz DK Langlois RA Memory CD8 T cells mediate severe immunopathology following respiratory syncytial virus infection. PLoS Pathog (2018) 14:e1006810. 10.1371/journal.ppat.1006810 29293660 PMC5766251

[119] Selin LK Varga SM Wong IC Welsh RM. Protective heterologous antiviral immunity and enhanced immunopathogenesis mediated by memory T cell populations. J Exp Med (1998) 188:1705–15. 10.1084/jem.188.9.1705 9802982 PMC2212518

